# Cost-effectiveness of atezolizumab versus chemotherapy in patients with non-small-cell lung cancer ineligible for platinum-based doublet chemotherapy

**DOI:** 10.3389/fpubh.2025.1349645

**Published:** 2025-05-02

**Authors:** Qiuji Wu, Yi Qin, Qiu Li

**Affiliations:** ^1^Division of Abdominal Tumor Multimodality Treatment, Cancer Center, West China Hospital, Sichuan University, Chengdu, China; ^2^Department of Radiation Oncology, Sichuan Clinical Research Center for Cancer, Sichuan Cancer Hospital & Institute, Sichuan Cancer Center, Chengdu, China

**Keywords:** atezolizumab, immunotherapy, cost-effectiveness, non-small-cell lung cancer, Markov model

## Abstract

**Background:**

Atezolizumab has recently demonstrated improved prognosis in patients with advanced or metastatic non-small-cell lung cancer (NSCLC) who are not eligible for treatment with a platinum-containing regimen, as observed in a randomized phase 3 clinical trial. This study aims to evaluate the cost-effectiveness of atezolizumab for the treatment of NSCLC from the perspective of payers in both developed and developing countries.

**Materials and methods:**

A Markov model was developed to simulate treatment scenarios involving atezolizumab or chemotherapy for patients diagnosed with NSCLC. The model estimated the transition probabilities, health care costs, and health utilities base on the risk of disease progression, survival, and toxicity using data from IPSOS clinical trials, relevant literature, and publicly available databases. A price simulation was conducted to guide the pricing strategy at the specified willingness-to-pay (WTP) threshold, and sensitivity analyses were performed to assess the model’s response to uncertainty.

**Results:**

Among patients with NSCLC who are not suitable for treatment with a platinum-containing regimen, the use of atezolizumab led to an incremental gain of 0.35 quality adjusted life years (QALYs) compared to chemotherapy. The ICER for atezolizumab compared to chemotherapy was calculated at $220400.53 per QALY in the US and $101874.61 per QALY in China. The price simulation results indicated that atezolizumab was favored in the US when the price was less than $371.28/60 mg and $474.92/60 mg at the WTP thresholds of $100,000 and $150,000, respectively; it was cost-effective at a WTP threshold of $36023.71when the price was about 40% of the current price in China. Sensitivity analysis revealed that variables such as the price of atezolizumab and utilities influenced the r model’s outcomes, although these factors did not significantly alter the overall conclusion.

**Conclusion:**

Atezolizumab was not considered cost-effective at the WTP thresholds of $150,000 per QALY in the US and $36,024 per QALY in China for patients with advanced NSCLC who are ineligible for platinum-based chemotherapy.

## Introduction

The global cancer data from the international Agency for Research on Cancer(IARC) indicated that lung cancer was among the most common cancer, contributing to a substantial number of new cases and deaths in 2020 (1). In the United States, lung cancer was estimated to have an incidence of 234,580 new cases and caused 125,070 deaths in 2024 (2). In China, approximately 828,100 new cases were reported in 2020 (3).The rapid advancement of emerging therapies, such as targeted therapies and immunotherapies, has notably improved survival rates for patients diagnosed with non-small cell lung cancer (NSCLC), the most prevalent form of lung cancer (4). Clinical trials investigating first-line immunotherapy for patients without targeted mutations primarily focus on individuals who can tolerate standard platinum-based chemotherapy, possess good performance status, and are relatively young (5). As researchers continue their quest for treatment alternatives surpassing chemotherapy in terms of both survival outcomes and quality of life, monotherapy with immune-checkpoint inhibitors (ICIs) is emerging as a promising approach.

Atezolizumab, a programmed death-ligand 1 (PD-L1) inhibitor, has shown improved overall survival compared to single-agent chemotherapy both in previously treated metastatic NSCLC patients (6, 7) and as first-line treatment in PD-L1–high NSCLC patients (8). Critical trials of first-line immunotherapy have been primarily limited to patients with good performance status, and there is limited evidence for its efficacy in patients with poorer performance status. Recently, the IPSOS trial investigated the safety and efficacy of atezolizumab versus chemotherapy alone in patients who were ineligible for treatment with a platinum-containing regimen (9). The findings indicated that initial treatment with atezolizumab significantly prolonged the median overall survival (OS) (10.3 vs. 9.2 months), increased the 2-year survival rate (24% vs. 12%), and maintained stabilization or improvement in quality of life, compared to chemotherapy alone (9).

Therefore, atezolizumab regimens appear to provide a viable therapeutic option for patients with NSCLC who are not eligible for treatment with platinum-containing regimens. However, given the substantial prevalence of NSCLC and the considerable number of advanced cases, the selection of a therapeutic agent will substantially impact the total cost of cancer treatment. According to a report from the National Cancer Institute, expenditure specifically on lung cancer increased from $21.1 billion in 2015 to $23.8 billion in 2020 (10). Additionally, the China Health Yearbook of 2022 reported that the average cost of hospitalization for lung cancer in China reached $5538.50 (11). Therefore, the economic impact of innovative drugs or new treatments should be considered in comprehensive assessments to guide the allocation of limited healthcare resources.

Several published studies have indicated that atezolizumab monotherapy or combination regimens may not achieve cost-effectiveness in both the US and China (12–15). However, the cost-effectiveness of atezolizumab monotherapy has not been assessed in patients with NSCLC who are ineligible for platinum-based chemotherapy. This study aimed to evaluate the cost-effectiveness of atezolizumab compared to chemotherapy for managing NSCLC cases ineligible for platinum-based doublet chemotherapy, from the perspectives of payers in both the US and China.

## Materials and methods

### Model construction

A mathematical model was developed to evaluate both the economic and clinical outcomes of patients with NSCLC who are ineligible for platinum-based treatment. The study compares the use of atezolizumab with single-agent chemotherapy by integrating decision trees and a Markov model. To simulate NSCLC progression, a three-health-state Markov model was constructed comprising progressive disease (PD), progression-free survival (PFS), and death (see Supplementary Figure S1). The model operates in monthly cycles over a 10-year time horizon, a duration chosen based on simulations demonstrating that over 95% of patients had died within this period. Transition probabilities among health states were derived from the IPSOS clinical trial. Patients entered the model in the PFS state, marking the beginning of their treatment, and the model’s endpoint was defined as patient mortality to reflect clinical reality.

The model was employed to estimate the total costs and effectiveness associated with each treatment option, with quality-adjusted life years (QALYs) serving as the measure of effectiveness. Subsequently, the incremental cost-effectiveness ratio (ICER) of atezolizumab relative to chemotherapy was calculated. This study was conducted from the perspective of payers in both developed and developing countries—represented by US payers (including public insurance, private insurance, and out-of-pocket payments) and the Chinese healthcare system, respectively (16). The willingness to pay (WTP) thresholds were set at $150,000 and $36023.71 per QALY, respectively, (17, 18). Annual discount rates of 3% for costs and 5% for utilities were applied (19, 20).

### Transition probabilities

This study employed a partitioned survival analysis to estimate transition probabilities among PFS, PD, and death states over time within the cohort. The probabilities for the states were determined by Kaplan–Meier (K-M) curves of OS and PFS from the IPSOS trial (9, 21, 22).Initially, outcomes points from the curves of OS and PFS were extracted using Plot Digitizer (version 2.6.8). Subsequently, the survival curves were reconstructed via the algorithm proposed by Guyot et al. using R statistical software (version 4.2.2; https://www.r-project.org/) (see Supplementary Figure S2) (23, 24). The reconstructed survival curves were then fitted to the Weibull, exponential, log-logistic, log-normal, gamma, Gompertz and generalized gamma distributions, respectively. The Bayesian information criterion (BIC), Akaike’s information criterion (AIC) and visual validation were employed to determine the best-fitting model. After evaluating the models’ fit, the log–normal model was selected to extrapolate the K–M curves beyond the IPSOS trial follow-up period (see Supplementary Figure S3). For details on the selected distributions and their application, refer to Supplementary Table S1. PFS and OS probabilities at time t were computed using Log-normal distributed survival functions. The proportion of patients in the PD state was calculated as the difference between the PFS and OS probabilities, while the proportion of patients in the death state was determined as 1-OS probability. Background mortality-representing the transitions from the PFS state to death, was estimated using age-specific life tables from the United States and China (25, 26).Microsoft Excel (Microsoft Corporation, Redmond, WA) was used to calculate the transition probabilities between states. The model was ultimately built and manipulated using TreeAge Pro software (Version 2020, https://www.treeage.com/).

### Cost and utilities

Direct healthcare costs considered in the model comprised drugs procurement, administration, best supportive care, adverse events management, and terminal care (Table 1). The cost of drug administration was based on the dosing regimen developed in the IPSOS trial: On days 1 of each 21-day cycle, atezolizumab was administered intravenously at a fixed dose of 1,200 mg, or single-agent chemotherapeutic drugs including gemcitabine (1,250 mg/m^2^ intravenously on days 1, 8, and 15 of a 28-day cycle) or vinorelbine (25 mg/m^2^ intravenously on days 1 and 8 of a 21-day cycle) were administered accordingly. Subsequent treatments and their associated costs were considered in the model. The model further incorporated a post-progression treatment regimen, including chemotherapy (pemetrexed), immunotherapy (Nivolumab), and best supportive care as subsequent treatments on a pro rata basis, based on the data provided in the IPSOS trial, and taking into account end-of-life costs (Supplementary Table S2). Additionally, the model accounted for grade 3/4 adverse events that exhibited significant differences between the study groups in the IPSOS trial. Notable events, such as neutropenia, anemia, dyspnoea, rash, nausea, and vomiting, were incorporated due to their clinical relevance (Supplementary Table S2) ($1 = ¥7.1368) (27).

**Table 1:** Model parameters and assumptions.

Parameter	United States		China	
	Model input (range)	Description and reference	Model input (range)	Description and reference
Drug cost, $				
Atezolizumab per 60 mg	620.83 (465.63–776.04)^b^	(36)	229.80 (172.35–287.25)^b^	(37)
Gemcitabine per 100 mg	13.22 (9.92–16.53)^a^	(36)	9.80 (0.79–133.95)^a^	(37)
Vinorelbine per 10 mg	42.21 (31.81–53.01)^a^	(36)	23.43 (7.71–49.59)^a^	(37)
Pemetrexed per 100 mg	282.7 (212.01–353.40)^a^	(36)	63.22 (5.28–111.82)^a^	(37)
Nivolumab per 10 mg	366.91 (275.18–458.64)^b^	(36)	129.61 (97.21–162.01)^b^	(37)
Cost of AEs, $ per unit^b^				
Dyspnoea	487.14 (365.36–608.93)	(38, 39)	119.10 (89.33–148.88)	Estimated
Anaemia	24530.87 (18398.15–30663.59)	(39–41)	7127.24 (5345.43–8909.05)	(18, 42)
Neutropenia	20802.80 (15602.10–26003.50)	(39–41)	953.00 (714.75–1191.25)	(18, 42)
Nausea	23418.14 (17563.61–29272.68)	(39–41)	90.61 (67.96–113.26)	(18, 43)
Rash	7834.14 (5875.61–9792.68)	(39, 44)	4119.71 (3089.78–5149.64)	(18, 42)
Vomiting	20461.36 (15346.02–25576.70)	(39–41)	90.61 (67.96–113.26)	(18, 43)
Others medical costs^b^				
Drug Administration per cycle	161.36 (121.02–201.70)	(39, 44)	47.64 (35.73–59.55)	Estimated
Follow-up and monitoring per cycle	193.00 (144.75–241.25)	(39, 45)	678.17 (508.63–847.71)	Estimated
Best supportive care per cycle	3104.19 (2328.14–3880.24)	(38, 39)	282.15 (211.61–352.69)	(18, 42)
Terminal care, one time	3143.94 (2357.96–3929.93)	(38, 39)	6883.05 (5162.29–8603.81)	(18, 46)
Utilities				
PFS	0.71 (0.5325–0.8875)^b^			(28)
PD	0.67(0.5025–0.8375)^b^			
Disutility due to AEs				
Dyspnoea	−0.05			(47)
Anaemia	−0.00			(44)
Neutropenia	−0.09			(48)
Nausea	−0.05			(48)
Rash	−0.03			(48)
Vomiting	−0.05			(48)
Discount rate, %	3 (0–5)			(49)

Atezo, Atezolizumab; Chemo, Chemotherapy; AE, adverse event; PD, progressed disease; PFS, progression-free survival.

a Local estimated.

b Range indicates 25% change.

The model employed health state utility (HSU) values to assign weights to survival time in each health state, thereby assessing the QALYs for different treatments. These HSU values reflect the overall well-being and functional status of patients across various health states. HSU values were assumed to be equivalent across treatment arms within the same health states, with those for PFS and PD derived from an observational cohort study (*N* = 263) that assessed health-related quality of life in advanced NSCLC patients using a validated EQ-5D questionnaire (28). Additionally, disutility values associated with adverse events (AEs) were incorporated into the model, derived from previously published research involving patients facing similar conditions as those in this study. Table 1 displays the HSU for the PFS and PD health states and the disutility values associated with AEs.

### Price simulation

We varied the price of atezolizumab per 60 mg between $0 and $700 to analyse the possibility of cost-effectiveness when the WTP threshold for the corresponding price is $100,000 or $150,000. In China, assuming a WTP equal to three times the GDP per capita, the estimated thresholds in 2023 were $84188.15 in Beijing (highest), $20121.20 in Gansu (lowest), and $36023.71 at the national level (18). Additionally, we varied the price between $0 and $250 to evaluate cost-effectiveness under the Chinese WTP thresholds of $20121.20, $36023.71, and $84188.15.

### Sensitivity analysis

Probabilistic sensitivity analysis (PSA) and one-way sensitivity analysis were conducted to assess the robustness of the model outcomes and conclusions in response to variations in key parameters. For the one-way sensitivity analysis, key parameters were varied based on their confidence intervals or by assuming a ±25% deviation from the base-case values. In the PSA, critical parameters such as cost and HSU data were incorporated. Costs were modeled using a gamma distribution, and HSU values were represented using a beta distribution. Subsequently, 1,000 simulations were performed utilizing the Monte Carlo simulation method.

### Subgroup analysis

In subgroup analyses, ICER was calculated using subgroup-specific OS and PFS hazard ratios (HRs) derived from IPSOS trial (9). Because PFS data by subgroup were not available for the Region classification, we assumed that these subgroups shared the same PFS HRs as the overall population. We evaluated the cost-effectiveness of patient subgroups defined by varying age, sex, Eastern Cooperative Oncology Group (ECOG PS) performance status scores, and PD-L1 expression levels. Due to insufficient data, proportional hazards were assumed. In addition to the base case analysis status, we also used simulated prices and specific WTP for subgroup analyses.

## Results

### Base case results

Base-case analyses were performed for both the US and China, and the detailed outcomes are presented in Table 2. In both countries, compared with chemotherapy alone, atezolizumab yielded an improvement of 0.5 life years (LYs) and 0.35 QALYs for patients with NSCLC ineligible for platinum-containing regimens. In the US, the total projected cost for the atezolizumab group was $132065.77, compared with an estimated $55221.04 for chemotherapy. This resulted in an ICER of $220400.53 per QALY gained. In China, the total anticipated costs were $54274.31 for atezolizumab and $18754.76 for chemotherapy alone, yielding an ICER of $101874.61 per QALY gained (see Table 2).

**Table 2:** Summary of cost and outcome results in the base-case analysis.

Scenario	Drugs	Cost ($)	Effectiveness (LY)	Effectiveness (QALY)	ICER, per QALY (vs Chemo, $)	Probability of cost-effectiveness (%)^a^
United States	Chemotherapy	55221.04	1.13	0.77		
	Atezolizumab	132065.77	1.63	1.12	220400.53	0.2%
China	Chemotherapy	18754.76	1.13	0.77		
	Atezolizumab	54274.31	1.63	1.12	101874.61	0%

ICER, incremental cost-effectiveness ratio; LY, life year; QALY, quality-adjusted life-year.

a Probabilities of cost effectiveness for Atezolizumab based on WTP threshold of $150,000 in US and $36023.71 in China, respectively.

### Price simulation

**Figure 1:**
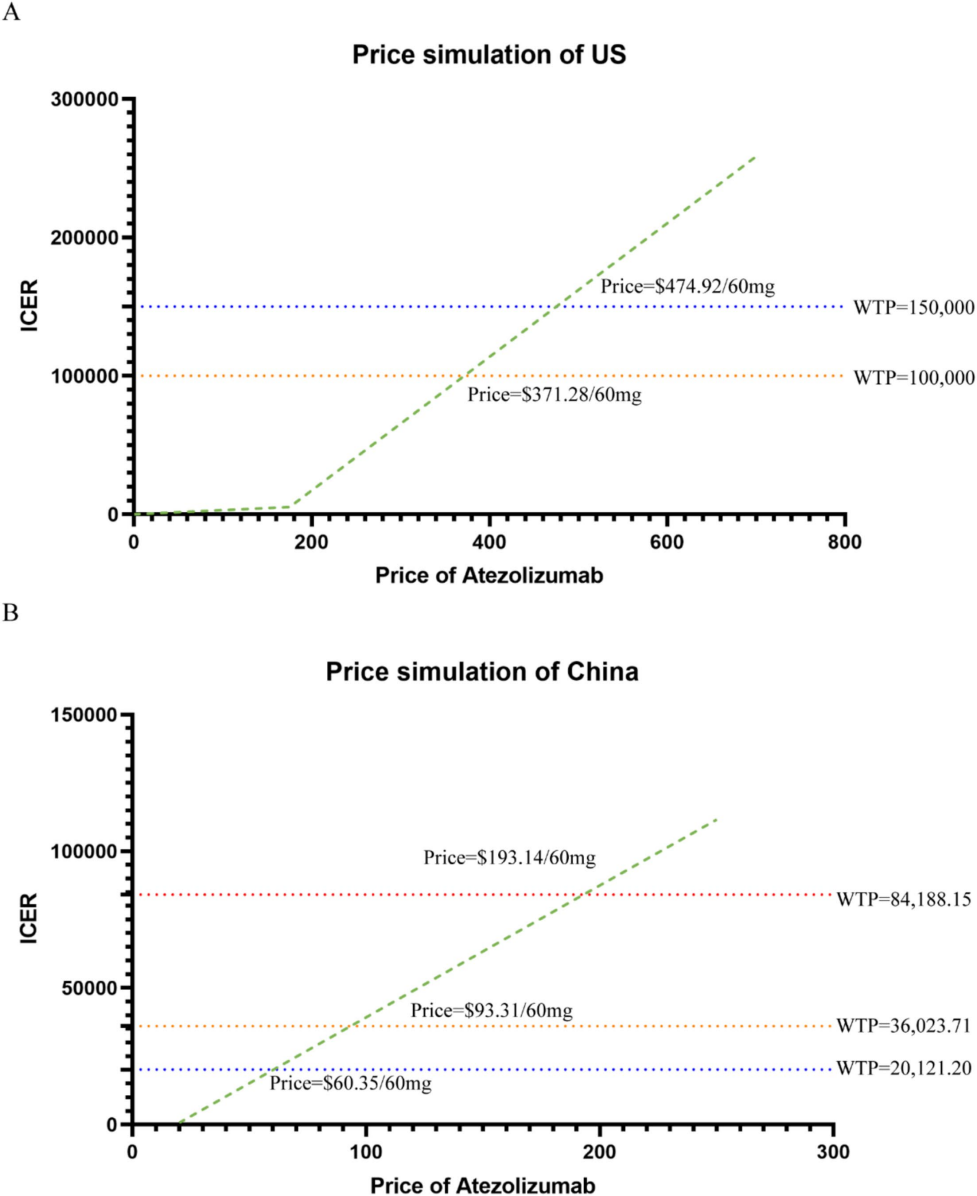
Results of price simulation and deterministic sensitivity analysis. **(A)** Dashed line perpendicular to the y-axis represents the given WTP, and the green dotted line represents the trend line of the ICER scatter point under each price in US. **(B)** Dashed line perpendicular to the y-axis represents the given WTP, and the green dotted line represents the trend line of the ICER scatter point under each price in China.

The results of the price simulation are presented in Figures 1A,B. In the US, when the price ranged from $0 to $700, the ICER increased as the cost of atezolizumab rose. Atezolizumab was considered cost-effective when priced below $372.28 per 60 mg and $474.92 per 60 mg, at the WTP thresholds of $100,000 and $150,000, respectively. In China, atezolizumab was considered cost-effective at prices of $193.14 per 60 mg, $60.35 per 60 mg, and $93.31 per 60 mg, corresponding to WTP thresholds of $84188.15, $20121.20, and $36023.71, respectively.

### Sensitivity analysis

**Figure 2:**
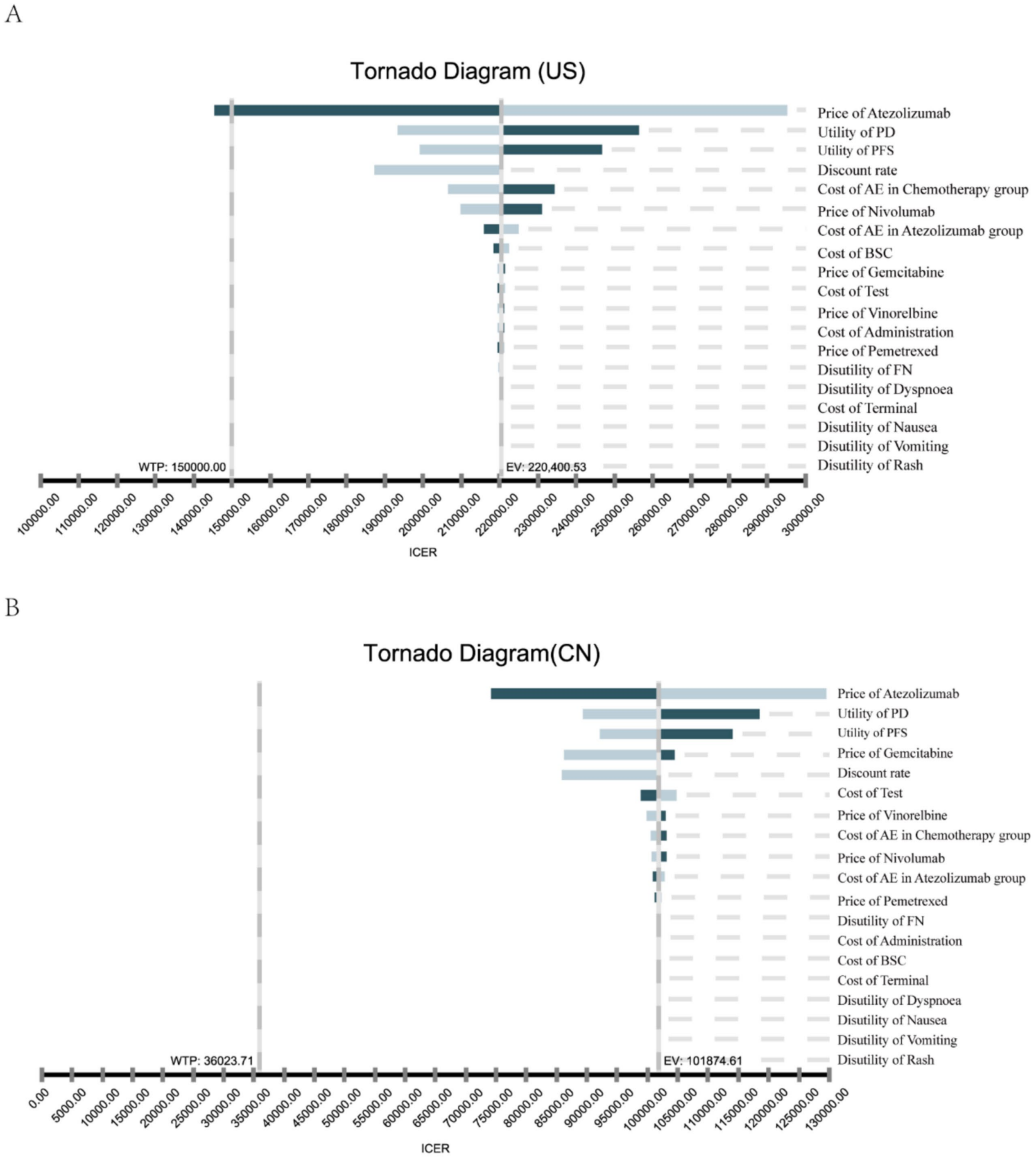
One-way sensitivity analysis. This diagram shows incremental cost effectiveness ratio (ICER) of atezolizumab vs. chemotherapy for different model input parameters of the United States **(A)** and China **(B)**. PFS, progression-free survival; PD, progression disease; AE, adverse events febrile neutropenia; BSC, best supportive care.

**Figure 3:**
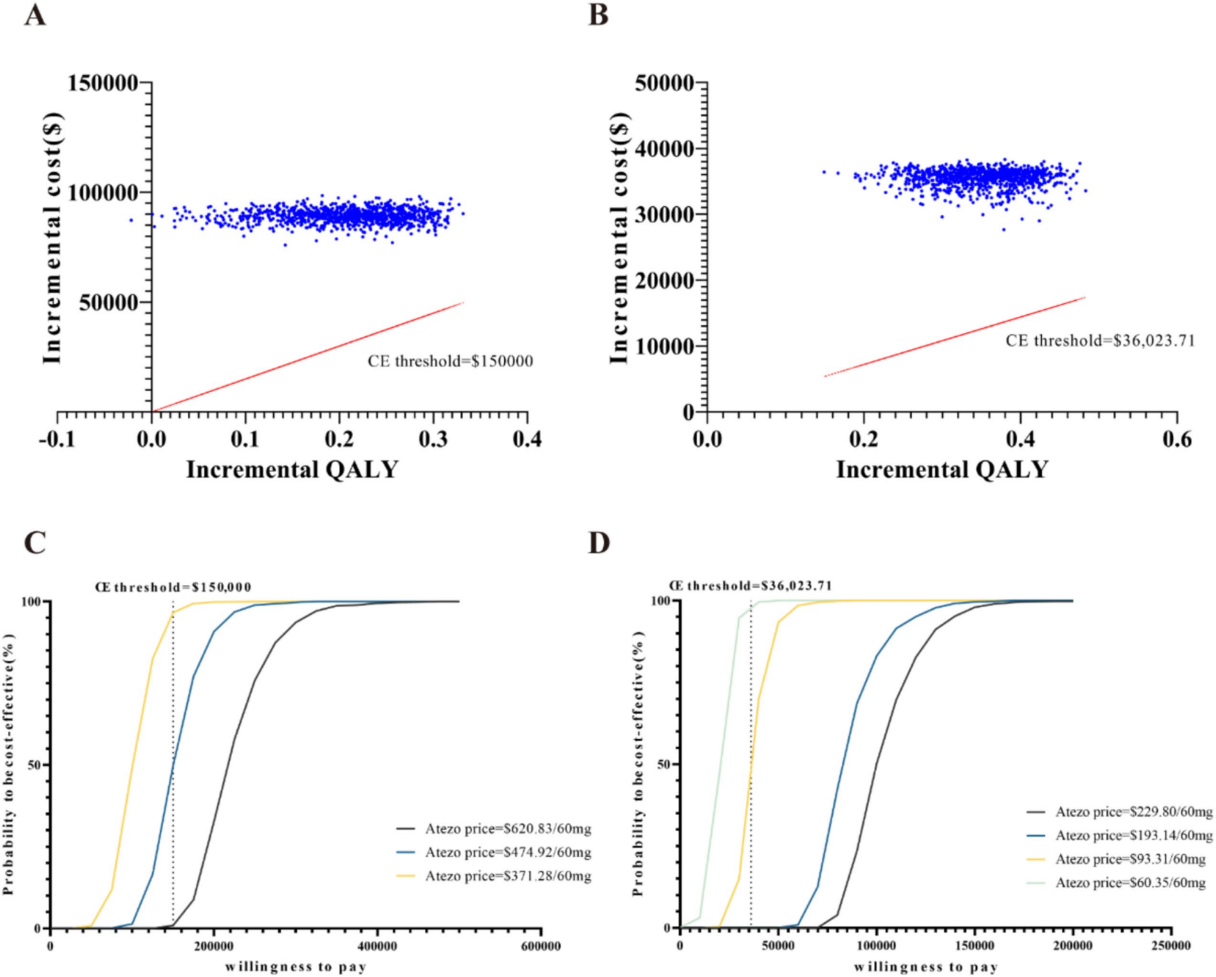
Probabilistic sensitivity analysis: scatter plot of the United States **(A)** and China **(B)**, cost-effectiveness acceptability curves of atezolizumab versus chemotherapy (1,000 iterations) of the United States **(C)** and China **(D)**. CE, cost-effectiveness; Atezo, atezolizumab; QALY, quality-adjusted life-years.

The results of the deterministic sensitivity analysis (DSA) are shown in Figures 2A,B. The base-case analysis results for both the US and China were most sensitive to the price of atezolizumab, followed by the utility values for PFS and PD states, discount rate, and chemotherapy-related AEs. According to Figure 2A, when the price of atezolizumab was below $474.92/60 mg, the ICER was less than the WTP threshold of $150,000 in the US. However, no variables were found to reduce the ICER below the WTP threshold of $36023.71 in China (Figure 2B). Through a PSA comparing the cost-effectiveness of atezolizumab to chemotherapy in both the US and China indicated that the probability of atezolizumab being cost-effective under the current WTP threshold was 0% (Figures 3A,B). The cost-effectiveness curve analyses conducted under both the base-case and simulated price scenarios are shown in Figures 3C,D. The cost-effectiveness acceptability curve provided a 0 to 57.3% probability of atezolizumab being cost-effective versus chemotherapy, at a WTP threshold of $150,000 to $225,000(1.5 × WTP) in the US. In China, the probability of atezolizumab being cost-effective ranged from 0 to 63.6% at WTP thresholds of $36023.71 to $108071.13 (3 × WTP). Moreover, as the price of atezolizumab decreased, the WTP required to achieve a 50% probability of cost-effectiveness also decreased in both the US and China (see Figures 3C,D).

### Subgroup analysis

Table 3 presents the results of the subgroup analyses. In most subgroups in the base-case analyses, the ICER was significantly influenced by the HR, with atezolizumab performing more favorably when the risk of death was lower. However, in all subgroups, the ICER for atezolizumab exceeded $150,000 per QALY in the US or $36023.71 per QALY in China compared with chemotherapy, suggesting an unfavorable cost-effectiveness profile for atezolizumab. Furthermore, simulated prices and various WTP thresholds were applied to assess the cost-effectiveness of atezolizumab across different subgroups (see Supplementary Table S3). In both the US and China, atezolizumab appeared to be favored among patients aged 70–79 years, those who never smoked or are current smokers, patients with Eastern Cooperative Oncology Group (ECOG) performance status scores of 0, 1, or 3, and patients with unknown PD-L1 status.

**Table 3:** Subgroup analysis results.

Subgroups	HR for OS	HR for PFS	ICER-US ($/QALY)	CE probability of Atezo^a^	ICER-CN ($/QALY)	CE probability of Atezo^b^
Age						
≥80 years	0.97 (0.66–1.44)	0.78 (0.53–1.14)	1944130.38 (131117.33-dominated)	0%	880864.13 (67091.62-dominated)	0%
70–79 years	0.68 (0.49–0.94)	0.89 (0.65–1.22)	177122.18 (73661.43–1244663.99)	22.1%	84741.60 (38461.4–554320.25)	0%
<70 years	0.75 (0.49–1.14)	0.87 (0.58–1.30)	243742.34 (70099.19-dominated)	0.1%	116489.88 (37203.39-dominated)	0%
Sex						
Male	0.76 (0.59–0.98)	0.76 (0.59–0.97)	287617.55 (119345.94–2547097.92)	0%	131945.71 (59443.69–1215802.33)	0%
Female	0.86 (0.58–1.27)	1.04 (0.7–1.52)	394018.43 (78833.48-dominated)	0%	194979.03 (43872.64-dominated)	0%
Race						
White	0.86 (0.67–1.11)	0.91 (0.70–1.17)	443519.00 (132842.60-dominated)	0%	213590.18 (95443.93–428534.02)	0%
Asian	0.74 (0.46–1.20)	0.75 (0.48–1.16)	265058.41 (70855.44-dominated)	0%	121414.82 (36548.51-dominated)	0%
Region						
Europe and Middle East	0.82 (0.63–1.08)	0.87 (0.70–1.07)	352734.36 (125010.67-dominated)	0%	166430.73 (62874.63-dominated)	0%
Asia Pacific	0.77 (0.47–1.26)	NA	268598.66 (77448.66-dominated)	0.1%	127095.72 (39172.30-dominated)	0%
Central or South America	0.73 (0.42–1.29)	NA	222489.14 (68054.55-dominated)	0.7%	105532.22 (34482.98-dominated)	0%
North America	0.79 (0.24–2.57)	NA	297671.46 (44892.93-dominated)	0%	140689.74 (22896.92-dominated)	0%
ECOG PS						
0 or 1	0.64 (0.36–1.13)	0.73 (0.42–1.28)	185517.18 (51964.37-dominated)	13.6%	83288.86 (27437.09-dominated)	0%
2	0.86 (0.67–1.10)	0.89 (0.70–1.14)	451821.33 (136044.09-dominated)	0%	213965.85 (69569.00-dominated)	0%
3	0.74 (0.35–1.57)	0.92 (0.44–1.93)	221049.70 (39461.52-dominated)	1.6%	106274.22 (22806.23-dominated)	0%
Tobacco use history						
Previous	0.83 (0.64–1.08)	0.85 (0.66–1.09)	382721.99 (127267.04-dominated)	0%	179428.15 (64312.38-dominated)	0%
Current	0.65 (0.40–1.07)	0.70 (0.42–1.15)	195050.93 (61432.84-dominated)	4%	88672.47 (31659.14-dominated)	0%
Never	0.70 (0.37–1.35)	0.91 (0.51–1.63)	187980.65 (45377.19-dominated)	15.7%	90327.09 (25310.47-dominated)	0%
Histology						
Non-squamous	0.77 (0.58–1.03)	0.86 (0.66–1.13)	271336.29 (101340.88-dominated)	0%	128024.64 (51798.00-dominated)	0%
Squamous	0.80 (0.58–1.12)	0.79 (0.57–1.10)	341590.46 (103711.49-dominated)	0%	157656.23 (52631.98-dominated)	0%
Stage						
IIIB	0.69 (0.39–1.24)	0.68 (0.38–1.21)	233023.98 (57847.92-dominated)	0%	105000.84 (30161.85-dominated)	0%
IV	0.81 (0.64–1.02)	0.86 (0.69–1.08)	335873.41 (128324.65-dominated)	0%	158105.91 (64683.19-dominated)	0%
Brain metastases						
Yes	0.85 (0.40–1.80)	1.14 (0.55–2.34)	334678.16 (38693.03-dominated)	0%	170527.86 (23607.19-dominated)	0%
No	0.78 (0.62–0.98)	0.82 (0.66–1.02)	297392.00 (126085.96–2552243.38)	0%	138631.68 (62640.52–1250122.18)	0%
Liver metastases						
Yes	0.94 (0.55–1.59)	1.31 (0.77–2.21)	776197.29 (54245.11-dominated)	0%	415624.60 (33835.39-dominated)	0%
No	0.77 (0.61–0.98)	0.77 (0.61–0.96)	298595.09 (128615.49–2545525.56)	0%	137285.23 (62949.10–1210177.47)	0%
Number of metastatic sites						
<3	0.74 (0.53–1.03)	0.76 (0.55–1.05)	262055.36 (92880.38-dominated)	0%	120385.12 (46657.72-dominated)	0%
≥3	0.78 (0.56–1.07)	0.91 (0.67–1.25)	271186.18 (87622.72-dominated)		129724.40 (46042.24-dominated)	0%
PD-L1 expression level						
<1%	0.81 (0.58–1.11)	0.90 (0.66–1.24)	322964.50 (93596.25-dominated)	0%	153798.31 (49072.38-dominated)	0%
≥1%	0.84 (0.62–1.15)	0.83 (0.62–1.12)	416622.72 (116060.96-dominated)	0%	194067.35 (59121.96-dominated)	0%
1–49%	0.84 (0.57–1.22)	1.01 (0.69–1.45)	350628.89 (79687.77-dominated)	0%	172130.84 (43698.50-dominated)	0%
≥50%	0.87 (0.50–1.52)	0.64 (0.38–1.08)	635250.97 (83386.87-dominated)	0%	279218.70 (42226.40-dominated)	0%
Unknown	0.49 (0.21–1.14)	0.41 (0.17–0.99)	173280.05 (44137.97–317318.32)	21.2%	73195.80 (22224.09–103573.72)	0%

CE, cost-effectiveness; ECOG PS, Eastern Cooperative Oncology Group performance status; HR, hazard ratio; ICER, incremental cost-effectiveness ratio; OS, overall survival; PFS, progression-free survival; PD-L1, programmed cell death ligand 1; US, United States; CN, China; Atezo, atezolizumab.

“Dominated” reveals that a plan is an absolute disadvantaged one.

a Probabilities of cost effectiveness for Atezolizumab based on WTP threshold of $150,000 in the US.

b Probabilities of cost effectiveness for Atezolizumab based on WTP threshold of $36023.71 in China.

## Discussion

The favorable outcomes associated with atezolizumab offer a viable therapeutic choice for patients with NSCLC who are ineligible for platinum-containing regimens, as it effectively delays disease progression (9). In this cost-effectiveness analysis, however, atezolizumab was not cost-effective as a first-line therapy for NSCLC patients compared with chemotherapy. The ICERs as high as $200,000/QALY in the US and nearly $100,000/QALY in China, both of which exceed the WTP thresholds.

The base-case model is most sensitive to the price of atezolizumab and the value of HSU, and adjusting the price of atezolizumab is more feasible in clinical practice than increasing the value of an HSU. To reduce the relatively considerable prices incurred by US patients, the US government has sought to align Medicare pharmaceutical prices with those paid by health systems in other developed countries (29). In China, there is an increasing trend towards the standardizing access negotiations for anticancer drugs within medical insurance frameworks, which is expected to become the primary pathway for incorporating innovative drugs into the medical insurance system. In 2019, negotiations for drug reimbursement in China covered 150 drugs, of which 97 reached agreements with the administration. Notably, within this group, 22 cancer drugs achieved average price reductions of 60.7 and 26.4% in their respective categories (30). However, our model indicates that a price reduction of 25% in the US and 60% in China would be necessary for the ICER of atezolizumab to fall below the respective WTP thresholds. Achieving such substantial price reductions presents significant challenges.

Following the publication of the IMpower110 study in 2020, numerous subsequent studies have investigated the cost-effectiveness of atezolizumab as a first-line treatment in patients with advanced or metastasis NSCLC who have favorable performance status and positive PD-L1 expression across various countries. Base-case ICER estimates range from a low of approximately $78,936 per QALY in China to a high of approximately $234,990 per QALY in the United States (13, 15, 31–33). In the US, specific studies have suggested that atezolizumab could be a cost-effective option for initial treatment in patients with high PD-L1-expressing metastatic NSCLC (31, 33). In contrast, several studies in China have indicated that atezolizumab might not represent a cost-effective solution (15, 31, 32). Moreover, due to differences in the study populations, the clinical benefit of atezolizumab compared to chemotherapy was less pronounced in the IPSOS trial than in the IMpower110 study. This suggests that further price reductions or modifications to charitable drug assistance programs may be necessary.

Another study by Jiang et al. analysed the cost-effectiveness of atezolizumab versus chemotherapy as first-line monotherapy in patients with non-small-cell lung cancer ineligible for platinum-based doublet chemotherapy (34). This analysis, also based on a Markov model but conducted in the United Kingdom, concluded that first-line atezolizumab monotherapy resulted in an additional 0.28 QALYs compared to chemotherapy monotherapy and was not considered to be cost-effective, with an ICER of £94,873 /QALY. These findings are consistent with our study conducted in the US and China. In contrast, Li et al. performed a similar analysis within the Chinese context and reported that atezolizumab was cost-effective in China (35). However, their study employed a fitted model only for the OS curve, without applying a similar fitting process to the PFS curve, and used fixed values to calculate the probability of transition to PFS, which may have further impacted the model results. Additionally, Li et al. appeared to have increased the proportion of immunotherapy in the follow-up treatment of the chemotherapy group to a greater extent, thereby directly contributing to the higher cost observed in the chemotherapy group.

Although our analysis primarily focuses on the US and China, the methodological framework and key findings offer insights that are applicable to other healthcare systems. Our examination of price simulations and cost-effectiveness acceptability curves demonstrates how fluctuations in drug prices and WTP thresholds influence cost-effectiveness outcomes. This suggests that healthcare systems in different regions can compare their local drug pricing and WTP thresholds with the scenarios we modeled. However, applying these findings to other contexts requires careful consideration of local healthcare settings, pricing structures, and demographic factors. Future research that adapts the model to specific regions will be essential for accurately assessing the cost-effectiveness of atezolizumab in diverse healthcare environments.

We acknowledge several limitations to our study. First, as the IPSOS trial individual patient data was inaccessible, the data regarding effectiveness and toxicity factors from reported studies were collected. In addition, the short-term clinical data were used to extrapolate long-term survival data from the IPSOS trial using log-normal models. Long-term survival benefits are inevitably subject to uncertainty. However, by comparing the trial curves with the simulated curves, we estimate that curve fitting and extrapolation had minimal impact on the results. Second, to simplify the analysis and enhance the generalizability of the results, we assumed that all patients in the IPSOS trial received proportional chemotherapy (Pemetrexed) and immunotherapy (Nivolumab) after progression, considering the costs of optimal supportive care and end-of-life care. Third, PFS was used as a surrogate for time on treatment in both treatment arms in the absence of available time on treatment data for chemotherapy and atezolizumab, introducing uncertainty regarding treatment duration. Finally, input data for treatment costs related to adverse events and the utility values for different states were not all from NSCLC patients. However, in the sensitivity analysis, we found that the variability of previous treatment costs, utility values, and AEs costs did not significant affect the outcomes.

## Conclusion

Our analysis indicates that the cost of atezolizumab for NSCLC treatment is exceptionally high and would not be considered cost-effective given the current prices of atezolizumab and WTP thresholds in the US and China.

## Data Availability

The original contributions presented in the study are included in the article/Supplementary material, further inquiries can be directed to the corresponding author.
