# Supplementary material for: Cost-effectiveness of atezolizumab versus chemotherapy in patients with non-small-cell lung cancer ineligible for platinum-based doublet chemotherapy
Source: Front Public Health. 2025 May 2;13:1349645. doi: 10.3389/fpubh.2025.1349645 (PMC12081414; doi:10.3389/fpubh.2025.1349645)
Supplement: Supplementary file 1 [file Data_Sheet_1.docx]

**
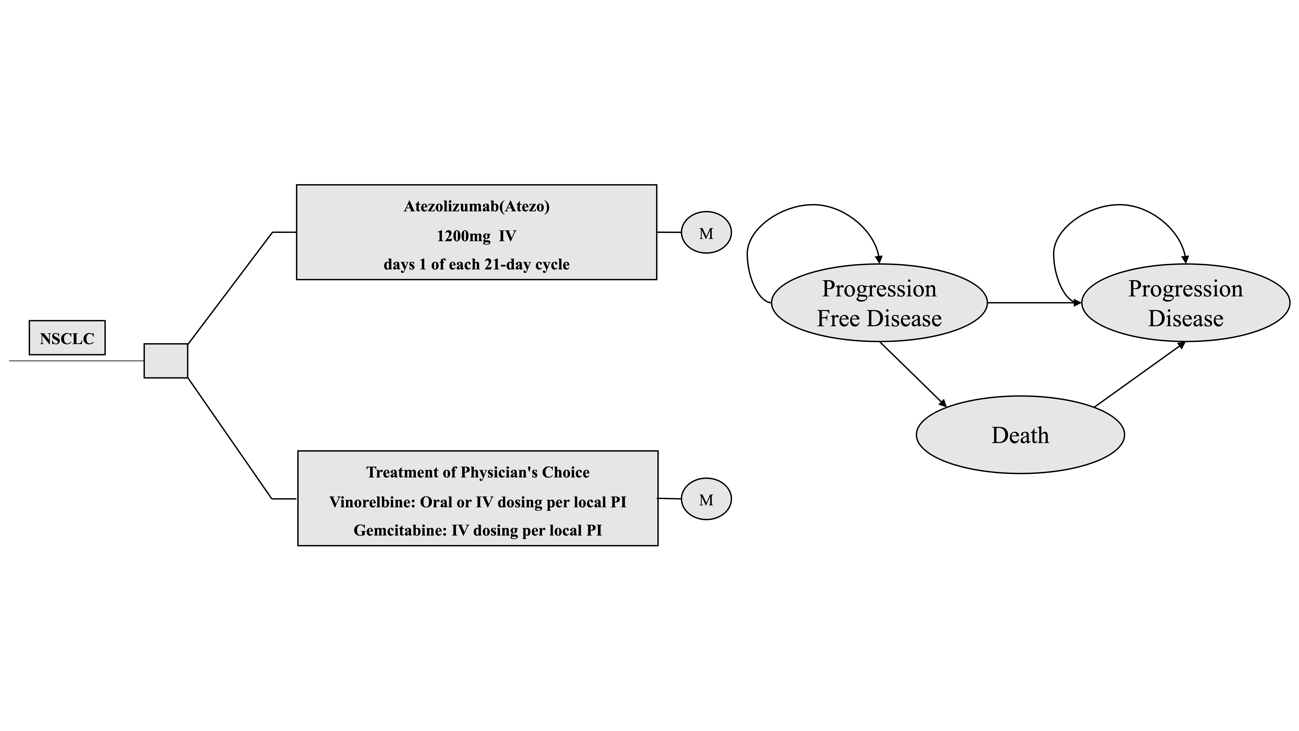
**

**Figure S1.** Abbreviated decision tree and Markov model used to compare two strategies for treating patients with non-small-cell lung cancer ineligible for treatment with a platinum-containing regimen and a network of three health states linked by transitional variables. PI, prescribing information; IV, Intravenous injection.


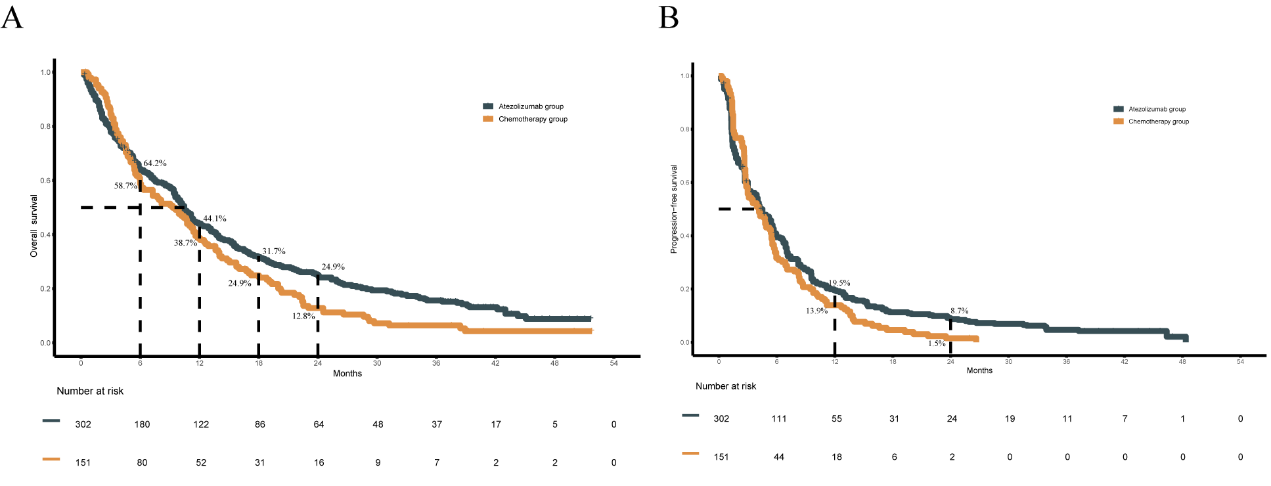


**Figure S2 The Reconstructed K-M curves of OS and PFS from the IPSOS trials**. (A) The Reconstructed K-M curves of OS between patients with Atezolizumab or chemotherapy , (B) The Reconstructed K-M curves of PFS between patients with Atezolizumab or chemotherapy .


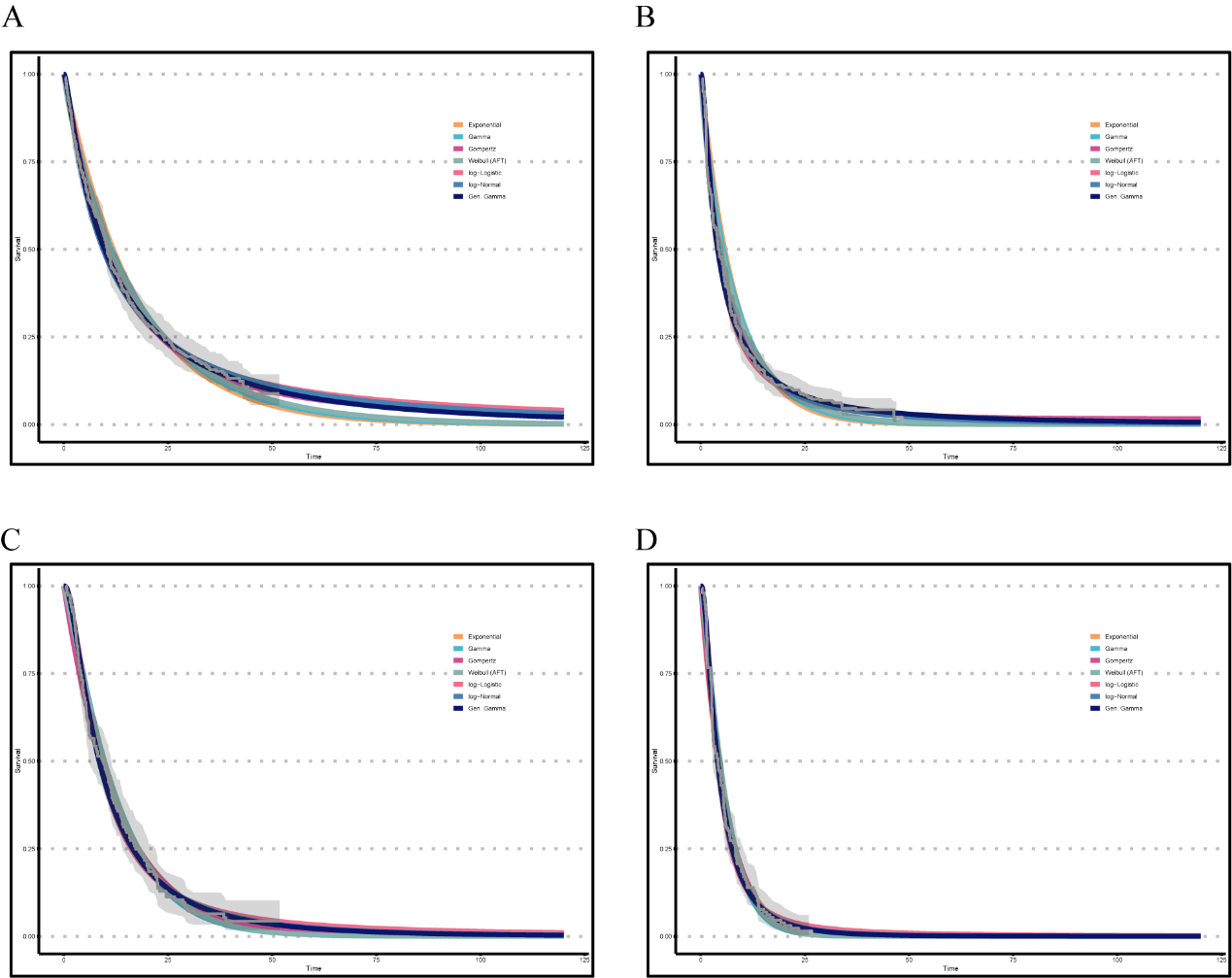


**Figure S3 Visual validation of models.** (A)Overall survival for atezolizumab, (B) Progression free survival for atezolizumab, (C)Overall survival for chemotherapy, (D) Progression free survival for atezolizumab.

**Table S1 Goodness-of-fit of parametric survival models**

| **Parameters** | **AIC** | **BIC** | **Value** |
| --- | --- | --- | --- |
| **Atezolizumab-OS** |  |  |  |
| Exponential | 1892.827 | 1896.538 | rate=0.0582422 |
| Gamma | 1892.130 | 1899.551 | Shape=0.8837612,rate=0.0506008 |
| Gompertz | 1884.240 | 1891.660 | Shape=-0.0195408,rate=0.0741189 |
| Weibull | 1889.844 | 1897.265 | Shape=0.894277,scale =16.770564 |
| log-Logistic | 1883.376 | 1890.797 | Shape=1.25545,scale =9.69185 |
| log-Normal | 1879.194 | 1886.615 | meanlog=2.24083,sdlog =1.36714 |
| Generalised Gamma model | 1879.855 | 1890.986 | Mu=2.389880, sigma=1.319384,Q=0.245343 |
| **Chemotherapy-OS** |  |  |  |
| Exponential | 922.264 | 925.281 | rate=0.0767722 |
| Gamma | 918.037 | 924.072 | Shape= 1.327484,rate=0.104086 |
| Gompertz | 924.263 | 930.298 | Shape=0.00012714,rate=0.07667309 |
| Weibull | 920.835 | 926.869 | Shape=1.13674,scale =13.42855 |
| log-Logistic | 911.909 | 917.944 | Shape=1.72097,scale =8.50021 |
| log-Normal | 908.376 | 914.411 | meanlog=2.133255,sdlog = 0.992844 |
| Generalised Gamma model | 910.316 | 919.368 | Mu=2.162954, sigma=0.988485,Q=0.062686 |
| **Atezolizumab-PFS** |  |  |  |
| Exponential | 1749.392 | 1753.102 | rate=0.119991 |
| Gamma | 1746.856 | 1754.277 | Shape=0.859353,rate=0.102341 |
| Gompertz | 1716.840 | 1724.260 | Shape= -0.0408031,rate= 0.1686612 |
| Weibull | 1738.740 | 1746.161 | Shape=0.858957,scale =7.748688 |
| log-Logistic | 1700.085 | 1707.506 | Shape= 1.40214,scale =4.18096 |
| log-Normal | 1691.172 | 1698.593 | meanlog=1.45120,sdlog = 1.21804 |
| Generalised Gamma model | 1690.952 | 1702.083 | Mu=1.300282, sigma=1.211649,Q= -0.25447 |
| **Chemotherapy-PFS** |  |  |  |
| Exponential | 790.384 | 793.401 | rate=0.166013 |
| Gamma | 782.520 | 788.554 | Shape=1.420104,rate=0.238459 |
| Gompertz | 791.875 | 797.910 | Shape=0.0115008,rate= 0.1560101 |
| Weibull | 786.476 | 792.511 | Shape=1.17263,scale =6.33342 |
| log-Logistic | 777.290 | 783.324 | Shape=1.85605,scale =4.02921 |
| log-Normal | 772.026 | 778.061 | meanlog=1.395637,sdlog = 0.922361 |
| Generalised Gamma model | 773.943 | 782.995 | Mu=1.425918, sigma=0.920075,Q=0.066931 |

AIC Akaike information criterion,BIC Bayesian information criterion,OS Overall survival ,PFS Progression free survival

**Table S2 Baseline values, ranges, and distributions of model parameters**

| **Parameters** | **Baseline Value** | **Low** | **Upper** | **Distribution** | **Source** |
| --- | --- | --- | --- | --- | --- |
| **Risk of grade 3–4 AEs** | | | | | |
| **Atezolizumab group** | | | | | |
| Dyspnoea | 5.00% | 3.75% | 6.25% | beta | ^1^ |
| Anaemia | 2.67% | 2.00% | 3.34% | beta | ^1^ |
| Neutropenia | 0.67% | 0.50% | 0.84% | beta | ^1^ |
| Nausea | 0.67% | 0.50% | 0.84% | beta | ^1^ |
| Rash | 0.67% | 0.50% | 0.84% | beta | ^1^ |
| Vomiting | 0.00% | 0.00% | 0.00% | beta | ^1^ |
| **Chemotherapy group** | | | | | |
| Dyspnoea | 4.76% | 3.57% | 5.95% | beta | ^1^ |
| Anaemia | 4.76% | 3.57% | 5.95% | beta | ^1^ |
| Neutropenia | 9.52% | 7.14% | 11.90% | beta | ^1^ |
| Nausea | 2.04% | 1.53% | 2.55% | beta | ^1^ |
| Rash | 0.00% | 0.00% | 0.00% | beta | ^1^ |
| Vomiting | 0.68% | 0.51% | 0.85% | beta | ^1^ |
| **Proportions of subsequent treatment** | | | | | |
| **Atezolizumab group** | | | | | |
| **Carboplatin** | 5.0% | 3.75% | 6.25% | beta | ^1^ |
| **Pemetrexed** | 5.0% | 3.75% | 6.25% | beta | ^1^ |
| **Gemcitabine** | 5.0% | 3.75% | 6.25% | beta | ^1^ |
| **Nivolumab** | 0.3% | 0.23% | 0.38% | beta | ^1^ |
| **BSC** | 80.0% | 60.00% | 100.00% | beta | ^1^ |
| **Chemotherapy group** | | | | | |
| **Carboplatin** | 5.0% | 3.75% | 6.25% | beta | ^1^ |
| **Pemetrexed** | 3.0% | 2.25% | 3.75% | beta | ^1^ |
| **Gemcitabine** | 1.0% | 0.75% | 1.25% | beta | ^1^ |
| **Nivolumab** | 9.0% | 6.75% | 11.25% | beta | ^1^ |
| **Atezolizumab** | 6.0% | 4.50% | 7.50% | beta | ^1^ |
| **Pembrolizumab** | 3.0% | 2.25% | 3.75% | beta | ^1^ |
| **BSC** | 70.0% | 52.50% | 87.50% | beta | ^1^ |

AEs adverse events,BSC best supportive care;

**Table S3 Summary results for subgroup analyses.**

| **Subgroups** | **US** | | | | **CN** | | | | | |
| --- | --- | --- | --- | --- | --- | --- | --- | --- | --- | --- |
|  | **ICER1^a^**  **^(range)^** | **CE probability** | **ICER2^b^**  **^(range)^** | **CE probability** | **ICER3^c^**  **^(range)^** | **CE probability** | **ICER4^d^**  **^(range)^** | **CE probability** | **ICER5^e^**  **^(range)^** | **CE probability** |
| **Age** | | | | | | | | | | |
| **≥80** | 794,446.00 | 0% | 1,267,757.95 | 0% | 107,028.32 | 3.5% | 257,552.85 | 0.2% | 713,464.98 | 0% |
| **70–79** | 84,563.46 | 72.9% | 123,003.18 | 77.9% | 21,895.03 | 36.7% | 34,119.78 | 61.6% | 711,46.38 | 76.5% |
| **<70** | 111,275.25 | 36.2% | 166,288.95 | 33.7% | 25,528.65 | 17.0% | 43,024.33 | 20.1% | 96,015.62 | 24.9% |
| **Sex** | | | | | | | | | | |
| **Male** | 133,306.17 | 9.5% | 197,423.40 | 5.3% | 27,206.29 | 10.2% | 47,579.93 | 5.8% | 109,288.07 | 2.8% |
| **Female** | 158,223.37 | 10.8% | 256,149.27 | 2.9% | 34,876.24 | 7.4% | 66,019.02 | 2.1% | 160,344.98 | 0.1% |
| **Race** | | | | | | | | | | |
| **White** | 185,959.33 | 3.6% | 292,924.09 | 0.2% | 36,391.89 | 6.6% | 70,409.25 | 1% | 173,441.79 | 0% |
| **Asian** | 124,494.58 | 14.9% | 182,870.87 | 13.4% | 25,973.19 | 13.4% | 44,538.25 | 11.7% | 100,768.51 | 11.7 |
| **Region** | | | | | | | | | | |
| **Europe and Middle East** | 153,768.33 | 5.8% | 236,399.10 | 1.7% | 31,334.53 | 8.0% | 57,613.09 | 1.4% | 137.206.20 | 0% |
| **Asia Pacific** | 120,984.92 | 21.1% | 182,289.04 | 17.9% | 26,767.28 | 11.3% | 46,363.45 | 9.6% | 105,413.92 | 7.2% |
| **Central or South America** | 102,949.14 | 48.2% | 152,594.21 | 48.5% | 24,365.59 | 20.5% | 40,153.91 | 29.5% | 87,973.94 | 46% |
| **North America** | 132,333.97 | 12.5% | 200,998.78 | 7.3% | 28,427.02 | 12.2% | 50,264.07 | 6.65 | 116,404.64 | 0.7% |
| **ECOG PS** | | | | | | | | | | |
| **0 or 1** | 90,308.80 | 63.6% | 128,187.72 | 77.3% | 21,359.16 | 40.9% | 33,405.56 | 67.2% | 69,881.99 | 83% |
| **2** | 190,669.80 | 1.6% | 299,126.26 | 0% | 36,646.24 | 5.9% | 71,137.99 | 0.9% | 175,607.39 | 0% |
| **3** | 100,698.18 | 50.7% | 150,680.27 | 52.5% | 24,556.58 | 23.7% | 40,452.08 | 32.7% | 88,596.74 | 44.1% |
| **Tobacco use history** | | | | | | | | | | |
| **Previous** | 166,687.68 | 3.6% | 256,406.88 | 0.4% | 32,742.80 | 7% | 61,275.65 | 1% | 147,696.60 | 0% |
| **Current** | 96,797.61 | 56.5% | 137,602.30 | 67.0% | 21,959.32 | 35.4% | 34,936.18 | 58.6% | 74.240.82 | 76% |
| **Never** | 88,367.28 | 65.0% | 129,678.33 | 71% | 22,622.58 | 33.9% | 35,792.28 | 54.2% | 75,680.99 | 69.3% |
| **Histology** | | | | | | | | | | |
| **Non-squamous** | 122,485.75 | 21.9% | 1084,303.52 | 17.2% | 26,956.41 | 11.5% | 46,615.94 | 10% | 106,167.17 | 6.7% |
| **Squamous** | 153,847.06 | 4.1% | 231,817.06 | 1.2% | 30,180.10 | 5.5% | 544,976.42 | 1.9% | 130,080.10 | 0.1% |
| **Stage** | | | | | | | | | | |
| **IIIB** | 113,556.13 | 26.4% | 163,171.24 | 31.0% | 23,883.21 | 20.0% | 39,662.00 | 29.9% | 87,453.16 | 42.5% |
| **IV** | 147,763.67 | 7.4% | 225,885.81 | 2.4% | 30,381.03 | 7.8% | 55,225.74 | 2.7% | 130,475.97 | 0.2% |
| **Brain metastases** | | | | | | | | | | |
| **Yes** | 131,192.72 | 25.4% | 215,700.40 | 13.2% | 32,363.01 | 11.5% | 59,238.48 | 5% | 140,639.50 | 0.6% |
| **No** | 134,602.41 | 10.1% | 202,209.07 | 7.5% | 28,098.96 | 9.7% | 49,599.50 | 4% | 114,720.81 | 0.9% |
| **Liver metastases** | | | | | | | | | | |
| **Yes** | 252,654.16 | 11.4% | 470,080.34 | 2.2% | 60,141.32 | 10% | 129,289.01 | 1.8% | 338,725.09 | 0.1% |
| **No** | 137,403.75 | 8.2% | 204,346.66 | 4.3% | 27,837.71 | 9% | 49,127.15 | 4.8% | 113,609.12 | 1.4% |
| **Number of metastatic sites** | | | | | | | | | | |
| **<3** | 122,865.75 | 18.4% | 180,671.31 | 16.4% | 25,876.56 | 12.1 | 44,260.12 | 12.3% | 99,940.66 | 14.9% |
| **≥3** | 120,274.61 | 24.8% | 182,948.32 | 19% | 27,256.76 | 15.1 | 47,188.50 | 9.1% | 107,558.21 | 7.7 |
| **PD-L1 expression level** | | | | | | | | | | |
| **<1%** | 140,588.17 | 11.3% | 216,329.22 | 3.4% | 30,067.09 | 10% | 54,337.16 | 2.6% | 127,846.87 | 0.2% |
| **≥1%** | 165,844.78 | 5.3% | 258,477.09 | 0.6% | 34,315.27 | 6.3% | 65,389.83 | 1.2% | 159,509.16 | 0.0% |
| **1–49%** | 144,632.88 | 14.6% | 230,182.21 | 4.9% | 32,261.34 | 9.8% | 59,468.39 | 2.4% | 141,873.73 | 0.0% |
| **≥50%** | 286,965.33 | 0.1% | 431,608.67 | 0% | 42,735.77 | 5.2% | 88,735.81 | 0.2% | 228,061.80 | 0% |
| **Unknown** | 92,805.97 | 70.2% | 125,915.36 | 78.9% | 19,063.99 | 62.3% | 29,593.57 | 80.3% | 61,485.79 | 90.5% |

CE cost-effectiveness,ECOG PS Eastern Cooperative Oncology Group performance status, HR hazard ratio, ICER incremental cost-effectiveness ratio, OS overall survival, PFS progress-free survival, PD-L1 programmed cell death ligand 1, QALY quality-adjusted life year, US United States, WTP willingness to pay.

^a^ICER1 is calculated based in US; price of atezolizumab = $371.28/60 mg; WTP = $10,000

^b^ICER2 is calculated based in US; price of atezolizumab = $474.92/60 mg; WTP = $15,000

^c^ICER3 is calculated based in China; price of atezolizumab = $60.35/60 mg; WTP = $20,121.2

^d^ICER4 is calculated based in China; price of atezolizumab = $93.31/60 mg; WTP = $36,023.71

^e^ICER5 is calculated based in China; price of atezolizumab = $193.14/60 mg; WTP = $84,188.15

1. Lee SM, Schulz C, Prabhash K, et al: First-line atezolizumab monotherapy versus single-agent chemotherapy in patients with non-small-cell lung cancer ineligible for treatment with a platinum-containing regimen (IPSOS): a phase 3, global, multicentre, open-label, randomised controlled study. Lancet 402:451-463, 2023
